# A green stabilizer for Nitrate ester-based propellants: An overview

**DOI:** 10.1016/j.heliyon.2024.e39631

**Published:** 2024-10-19

**Authors:** Siti Nor Ain Rusly, Siti Hasnawati Jamal, Alinda Samsuri, Siti Aminah Mohd Noor, Khoirul Solehah Abdul Rahim

**Affiliations:** a Centre for Defence Foundation Studies, National Defence University of Malaysia, Malaysia; b Centre for Tropicalization, National Defence University of Malaysia, Malaysia

**Keywords:** *Nitrate ester*, *Green propellants*, *Stabilizer*, *Stability*, *Nitrocellulose*

## Abstract

The field of propellants has recently witnessed dynamic shift, including advancements in propulsion technology and a growing emphasis on the development of environmentally friendly propellants. Nitrate ester (NE) are extensively used in solid propellants, exhibiting chemical instability as they undergo decomposition reactions. Stabilization is a crucial aspect in propellant, ensuring the safety and reliable performance of energetic materials. Stabilizer plays a vital role in inhibiting or slowing down the autocatalytic decomposition reaction of propellants. In response to grow health and environmental concerns, there is a continuous effort to explore and evaluate green stabilizers designed to replace traditional stabilizers, which have been associated with adverse environmental impacts. Therefore, this study aimed to provide an overview of the current research carried out in the field of NE-based propellants, emphasizing the most significant work undertaken on green stabilizer materials for NE-based propellants. A comprehensive review of various environmentally friendly and low-toxicity stabilizers employed in propellants are presented, and their effects on the stability and shelf-life performance of NE-based propellants are discussed. Furthermore, this paper delves into the stabilization mechanisms of green stabilizers to mitigate decomposition reactions, thereby preventing unwanted side effects and ensuring long-term storage stability. Through a comprehensive review of recent developments, the manuscript highlights the successes and challenges associated with the incorporation of green stabilizers in NE-based propellants formulations. Finally, the review concludes by outlining future research directions and opportunities for innovation in sustainable and green stabilizers as well as key issues that need to be addressed and resolved. The comprehensive review and insights provided in this study contribute to the ongoing efforts in developing safer and more sustainable propellant technologies.

## Highlights

• Recent advancements in propulsion technology have led to a shift towards environmentally friendly propellants.
• Nitrate ester (NE) in solid propellants are chemically unstable, necessitating stabilization for both safety and performance.
• This study highlights the significance of green stabilizers as alternatives to conventional stabilizers, considering environmental impacts.
• The review discusses green stabilizers in propellants, their effects on stability, shelf-life, and the stabilization mechanisms.
• The insights support efforts to develope safer and more sustainable propellant technologies.

## 1 Introduction

The current trend in propulsion field is geared towards adopting environmentally friendly propellants, necessitating the exploration and rapid development of green alternatives to overcome existing drawbacks by traditional propellants. These drawbacks include significant environmental risks due to their toxicity, operational handling challenges, and adverse impacts on ecosystems, including the release of harmful substances and unburnt particles during combustion [1]. The development of environmentally friendly propellants holds significant importance in preserving the environmental well-being, especially with the rapid expansion of the energetic materials industry. Therefore, there is significant research focus on environmentally friendly green energetic materials over worldwide [2]. As evident, various global entities are actively contributing to this research such as projects like Green Advanced Space Propulsion (GRASP), Pulsed Chemical Rocket with Green High-Performance Propellants (PulCheR), and Green Propellant Infusion Mission (GPIM) technology by NASA [3].

**Fig. 1:**
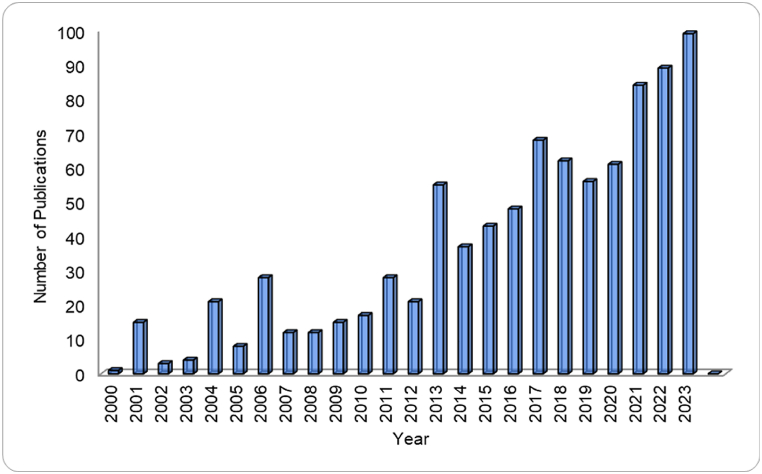
Number of scientific publications using the search terms ‘‘green propellant” since 2000 in Scopus database on July 2024 (Scopus website). (For interpretation of the references to colour in this figure legend, the reader is referred to the Web version of this article.)

Green propellants are broadly interpreted as low hazard, low toxicity and environmentally friendly attributes throughout different stages of propellants development, operations and storages compared to traditional toxic propellants [4]. The green propellants have demonstrated significant advantages not only in terms of operational efficiency and environmental safety but also contribute to cost-effectiveness and higher commercial value by reducing associated costs in transportation, storage, handling, and operations since traditional toxic propellants such as hydrazine cause high storage and handling costs [5]. Accordingly, advances progress has been made in the development of green propellants and ongoing research in this field remains highly dynamic as shown in Fig. 1.

**Fig. 2:**
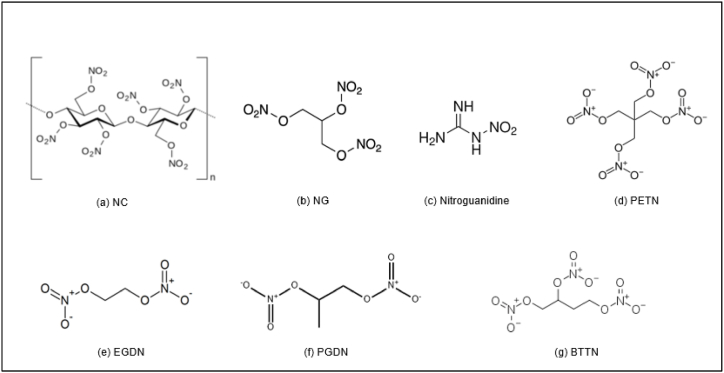
Chemical structure of nitrate ester.

Nitrate ester (NE) are energetic materials that possess explosive properties and often used as explosives and propellants due to their high energy content. NEs are formed by the reaction between a nitric acid and an alcohol, involving the substitution of hydrogen atoms in the alcohol's hydroxyl groups with nitrate groups. Specifically, it involves the substitution of the hydrogen atoms in the alcohol's hydroxyl (-OH) groups with nitrate (-NO_3_) groups. Common examples of NEs (Fig. 2) include nitrocellulose (NC), nitroglycerine (NG), nitroguanidine, pentaerythritol tetranitrate (PETN), tri-ethylene glycol dinitrate (EGDN), 1,2-propyleneglycol dinitrate (PGDN), 1,2,4-butanetriol trinitrate (BTTN) [6,7] are extensively employed in various civilian and military applications in many years. NEs play a crucial role in the development of explosives and propellants due to their ability to release a significant amount of energy upon decomposition or combustion. However, propellants that contain NE known as NE-based propellants is inherently chemically unstable and experience decomposition even when stored under standard storage temperature conditions [8]. The chemical instability of NE-based propellants is influenced by the chemical structure of the explosive, with aliphatic nitrate esters and structural properties, such as hydrogen bonding and packing structures, which can lead to sterically hindered planes and affect stability [9]. In the absence of stabilizer, the instability of NE can lead to autocatalytic decomposition, self-heating and safety hazards that affecting both the service life and the ballistic performance.

The stabilizer has the capacity to scavenge free radicals or reactive oxygen species, subsequently disrupting chain reactions that propagate decomposition. As a result, it hinders further degradation of the substance, leading to a deceleration in the decomposition rate [8,10]. The most commonly utilized stabilizers are from the classes of aromatic amines (e.g: diphenylamine (DPA)) and aromatic urea derivatives (akardite and centralite). Despite their high stabilizing capabilities, conventional stabilizers generate carcinogenic substances at certain stages during the propellant's service life. Stucki (2004) [11] reported that N-nitroso-DPA is a major degradation product in the stabilization process of propellants by DPA, and has been found to possess carcinogenic and toxic properties. This finding has been supported by a research study conducted by Rodrigues et al. [12]. Additionally, urea-based stabilizers such as ethyl centralite and akardite-II, lead to the formation of N-nitroso amides that contain the N-nitroso group, which is recognized to have carcinogenic, mutagenic and reprotoxic properties [13,14]. All of these studies highlight the potential health risks associated with the degradation products of propellants containing conventional stabilizers. Thus, further research is needed to explore the replacement of conventional stabilizers with environmentally friendly alternatives. The scientific community aim to mitigate or eliminate the presence of these harmful substances, prioritizing the preservation of environmental and human health. Advances progress has been made in the development of new stabilizers with a focus on identifying effective stabilizers as well as have less adverse effect on environmental and health issues [[15], [16], [17]].

As such, new stabilizers that are safer and more sustainable have attracted more attention from the researchers worldwide. The use of green stabilizers would greatly reduce the risks associated with toxicity and hazardous contamination of the environment. In general, green stabilizer refers to a stabilizing agent employed in propellants, where the term "green" signifies its alignment with environmentally friendly and sustainable practice in which it specifically designed to reduce or mitigate the environmental and toxicological hazards associated with existing stabilizers. A green stabilizer not only contributes to the stability and safety of energetic materials but also adheres to principles of environmental responsibility and resource efficiency [18]. This type of stabilizer can encompass either naturally occurring substances or synthetically derived compounds. Several studies have suggested replacing conventional stabilizers with natural products, particularly those derived from sustainable sources which are abundantly available and suitable for large-scale production [16,17,19]. In this context, Fryš et al. have investigated natural product derivatives such as deoxidized soy bean oil, deoxidized linseed oils and deoxidized mixture of fatty acids as new non-toxic stabilizers for NC-based propellants and smokeless powders [20,21]. Additionally, Krumlinde et al. have identified phenol bis (2,6-dimethoxyphenyl) triethyleneglycol as a promising chemical stabilizer that prevents the formation of by products containing nitrosamines [22].

One notable study investigated the use of mordenite zeolites as a stabilizer for NC [23]. In another study, zeolites which are an inorganic non-toxic compound also have been explored as new stabilizer by Zayed et al. [24]. This suggests that zeolites can be an effective and safer alternative stabilizer for NC-based formulations. Other natural substances that have been investigated are homopolymers and copolymers derived from lignin [25,26], guaiacol [17] and curcumin [13]. Moreover, Dejeaifve et al. conducted a study on five types of green molecules such as 2,3,5-trimethylphenol, 1,2,3-trimethoxybenzene, curcumin, α-ionone, and α-tocopherol; and found that these molecules exhibit stabilization properties for nitrate ester [27]. The efficiency of these green stabilizers was comparable to or even better than conventional industrial solutions such as DPA and akardite II [28]. For instance, the heat flow of double base powders containing α-ionone remains stable over time, with autocatalysis occurring 1.5 to 4 times slower compared to powders stabilized with DPA [29,30].

Fullerene derivatives have shown significant potential as stabilizers in NEs compounds [29,31]. The application of fullerene-based stabilizers is primarily due to their ability to scavenge nitrogen oxide free radicals produced during NEs degradation, which effectively eliminates nitroxide radicals and inhibits the autocatalytic decomposition that causes NEs instability [32,33]. These derivatives exhibit high stability and resistance to thermal decomposition, making them promising candidates for enhancing the safety and performance of energetic compositions [[32], [33], [34]]. Research on fullerene-based stabilizer aims to develop innovative solutions that enhance the safety of handling and storing energetic materials by preventing undesired decomposition reactions while minimizing negative environmental and health impacts. Additionally, the fullerene-based stabilizer was found to be more stable than traditional stabilizers and maintaining good performance even at high temperatures. This stabilizer changed the decomposition mechanism of NC from a self-accelerating catalytic model to a non-autocatalytic reaction model. Specifically, the fullerene stabilizer had a nitroxide radical scavenging efficiency of 73.4 %, effectively preventing acidity changes caused by the thermal decomposition of NC [35].

In recent years, numerous review papers have extensively explored energetic materials, propellants and their components, such as oxidizers, binders and stabilizer [36,37] as summarized in Table 1. In particular, there has been a significant focus on green technology, with various reviews covering topics like green energetic materials [38], green monopropellants [4] and green oxidizers [39]. Although stabilizers for NE-based energetic materials have been addressed by Trache et al. [40] and Gańczyk-Specjalska [41], to the best of our knowledge, there is currently no review paper specifically focusing on green stabilizer for propellants. Owing to the rapid ongoing research activities in green propellants, there is a need to thoroughly study and compile various data on the properties and performance of green stabilizers. Therefore, this paper aims to provide an overview of current research on NE-based propellants, emphasizing significant work related to green stabilizer materials for NE-based propellants. This paper stands out with comprehensive discussions on green stabilizers of NE-based propellants, encompassing synthesis and systematic evaluations of their characteristic properties. By evaluating existing studies and reviews, this paper identifies gaps and areas for future research, guiding ongoing and future efforts in the development of green propellant technologies. Hence, this review makes a valuable contribution by offering current overview of green stabilizers in enhancing the performance of propulsion systems and reducing environmental impact.

**Table 1:** Previous related review papers related with development of energetic materials and propellants.

Year	Objective/Highlight	Focus remarks	Reference
2009	This study presents existing literature on Green Energetic Materials (GEMs) by focusing the global efforts, initiatives, and principles shaping the development of environmentally friendly and high-performance of energetic materials.	Green energetic materials	[38]
2015	This article presents a comprehensive review of the characteristic properties of oxidizers/energetic fillers utilized in composite propellant. The advantages and disadvantages of these ingredients for specific and potential propellant applications are also discussed.	Oxidizers/energetic filler	[37]
2017	This review highlights recent progress on green oxidizers including their production and characteristics properties in order to replace AP in solid propellants.	Green oxidizers for solid rocket propulsion	[39]
2017	This review paper provides an overview and summary of existing and emerging stabilizers, along with an analysis of their mechanisms of action. It critically and analytically evaluates both the advantages and drawbacks associated with these stabilizers.	Stabilizers for nitrate ester based energetic materials	[40]
2018	This review offers detailed overview on the physical and chemical properties, thermal decomposition, and combustion behavior of ADN and ADN based propellants. Catalytic effect on thermal decomposition, combustion wave structure, and burning rate of ADN is also discussed.	Ammonium dinitramide (ADN) and ADN based solid propellants	[42]
2019	This paper aims to find better or equivalent alternative stabilizers for nitrocellulose propellants by conducting analyses such as chromatography, stability assessments, and thermochemical evaluations	Nitrocellulose stabilizers used in gun propellants	[41]
2019	This paper focuses on developing phase stabilized ammonium nitrate (AN) and AN/Potassium dinitramide (KDN)-based green oxidizers as an alternative to replace toxic ammonium perchlorate (AP) and hydrazine in solid and liquid rocket motors.	AN and AN/KDN based-green oxidizers	[3]
2020	A comprehensive review of various rocket propellant types, including solid, chemical, and green propellants. This paper presents a systematic evaluation to facilitates the prediction of the most suitable fuel for future rocket engines.	Materials and characterization of rocket propellant	[43]
2021	This review offers a comprehensive examination of nitric esters, delving into their molecular structures, physical properties, and their performances as energetic materials. Additionally, it discusses newly developed materials, including the synthetic protocols for their production.	Nitric Ester by focusing their roles in energetic materials.	[44]
2021	This review provides an overview of green monopropellants and explores various propulsion system configurations	Green monopropellants	[4]
2023	This paper explores nitrate ester binders including recent advances of energetic binder materials by emphasizing the synthesis, properties, and their applications in solid propellants.	Binders for nitrate ester	[36]

## 2 Solid Propellant

**Fig. 3:**
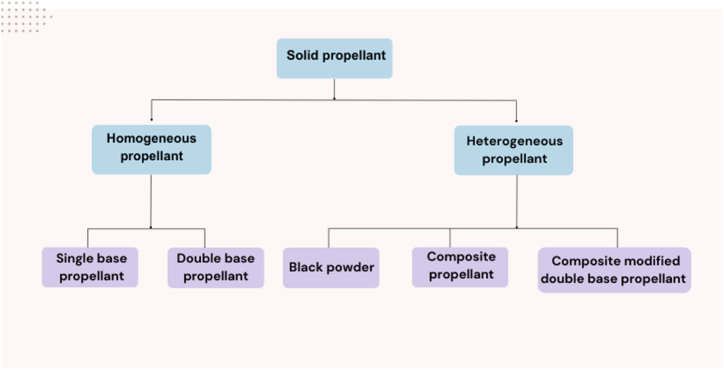
Classification of solid propellant [52].

Propellants are composed of highly energetic materials capable of generating high-temperature gaseous products, which are used to achieve propulsive forces. Solid propellants are utilized across a wide range of civil and military applications, including rocket engines, spacecraft, small ammunition and missile system [[45], [46], [47]]. Solid propellants offer several advantages, including simplicity for cost-effective maintenance and savings in high-production-rate systems, exhibit long storage stability and are resistance to unintended detonation [48,49]. Their simplicity also ensures reliability and provides higher mass flow rates during launch compared to liquid propellants. Furthermore, solid propellants can be tailored to meet specific requirements, such as high combustion rates, energy values, and safety performance, making them suitable for various tactical and strategic weapon systems. They are categorized based on the number of NEs present in the propellant's chemical composition. These categories include single-base (SB), double-base (DB), and composite propellants, as depicted in Fig. 3 [[50], [51], [52]]. Each type is designed to meet specific performance and safety requirements, further enhancing their versatility and applicability in diverse fields.

SB propellants are made up of a single energetic compound, primarily consisted of nitrocellulose (NC) present in the composition from 85 up to about 96 % [53]. SB propellants are often used in small caliber ammunition and rocket motors for missiles and other small rockets. In comparison to other propellant types, SB propellants exhibit a relatively lower energy density, resulting a less thrust per unit of mass. In the case of DB propellants, it contains a mixture of NC and nitroglycerine (NG) or other NEs compound like nitroglycol, (EGDN). NC is the primary component, providing the fuel for combustion, while NG/EGDN is the plasticizer that helps to control the burn rate of the propellants. The addition of NG/EGDN enhances the propulsion performances of propellants and provides a higher energy density, making it a popular choice for rocket and missile propellants [53]. DB propellants are formulated in response to the military's need for extended shooting distances, aiming for higher bullet velocities and increased propellant energy. This propellant type incorporates nitroglycerine (NG) or EGDN as energetic plasticizers to NC [53]. The solventless production technology employed in double-base (DB) propellants allows for the creation of larger propellant grains with large wall thickness and complex geometries, meeting the requirements for enhanced performance. However, DB propellants are more sensitive to heat and impact, and require more careful handling and storage to ensure safety. Additionally, a combination of DB propellants with nitroguanidine is classified as triple-base propellants [54]. Since nitroguanidine has a relatively high mole fraction of hydrogen in its molecular structure, the molecular mass of the combustion products decreases, even when the flame temperature is lowered.

Solid propellants can be mixed with various chemical constituents known as additives such as oxidizers, polymeric fuel, plasticizer, curing agent, and stabilizer to modify or improve their features [45,55]. Each constituent plays a specific role in enhancing performance, stability, and efficiency of the propellants as summarized in Table 2. Understanding the function of these constituents is essential for optimizing propellant formulations and improving their performance. The composition of solid propellants is tailored based on desired combustion characteristics for specific applications like space launches, missiles, and guns due to variations in chemical ingredients and proportions yielding diverse physical and chemical properties, combustion traits, and overall performance. In composite solid propellants, oxidizers act as catalysts, facilitating an exothermic, heterogeneous reaction between the solid fuel and oxidizer decomposition products, thereby enabling the efficient and safe combustion of the fuel [56]. The polymeric fuel in solid propellants serves dual functions, acting as binders for oxidizer particles and as fuel, ensuring structural integrity, fuel provision, and lubrication [57]. The addition of plasticizers into solid propellants improves both the mechanical and processing properties of the binder [58,59]. The curing agent, also referred to as a crosslinker, significantly influences the physical properties, manufacture properties, and aging of composite propellants, ultimately solidifying and hardening the binder [60,61]. Meanwhile, stabilizers in solid propellants play a crucial role in preventing early decomposition or explosion during storage. They inhibit the ongoing decomposition of nitrate ester, enhancing chemical stability and contributing to the modification of ballistic properties in the propellants [[62], [63], [64]].

**Table 2:** The representative functional components of solid propellants and their suggested formulation [48].

Component	Main Function	Amount %
Oxidizer	Catalysts for efficient and safe combustion of the fuel.	60–85
Polymeric fuel	Acts as binders for structural integrity and provides fuel, lubrication.	8–10
Plasticizer	Improves mechanical and processing properties of the binder.	2–10
Curing Agent	Solidifies and hardens the binder, influencing physical properties and aging.	1
Stabilizer	Prevents early decomposition or explosion, enhances chemical stability, and modifies ballistic properties.	1–5

Solid propellant technology has advanced significantly in recent years, driven by the need for higher performance, environmental sustainability, and safety, resulting in the development of insensitive munitions that are resistant to accidental ignition [65]. Recent research has focused on improving performance, safety, and environmental impact through advancements in materials, formulations, and combustion technologies [58,[66], [67], [68]]. Modern binders, such as energetic plasticizers and high energy density materials, are being developed to enhance performance, increase energy output, and improve the safety of solid propellants by reducing sensitivity [60]. Additionally, the use of nanoscale combustion catalysts, including metal oxides, metal powders, and metal alloys, has significantly improved the burn rate and overall combustion efficiency of composite solid propellants [45]. Energetic Metal Organic Frameworks (EMOFs) are being investigated for their potential to act as effective burning rate catalysts, with research focusing on monometallic, bimetallic, and carbon-supported [[69], [70], [71]]. The use of green oxidizing agents in propellant formulations has been highlighted for their potential in boosting propellant performance while addressing environmental concerns [3,72,73]. Furthermore, advancements in process technology, utilizing new energetic materials such as Ammonium Perchlorate (AP), Hydroxyl Terminated Polybutadiene (HTPB), Aluminum (Al), and Cyclotrimethylene Trinitramine (RDX), have shown promise in developing smokeless propellants with enhanced propulsion performance and reduced environmental impact. These AP/HTPB/Al-based and RDX composite propellants use AP and RDX as an oxidizing agent, HTPB as a binder, Al as a high-energy fuel, and Toluene Diisocyanate (TDI) as a curing agent [74]. These developments signify a shift towards more efficient, safer, and environmentally friendly solid propellant technologies.

The extensive studies on green propellants are numerous and aims to develop environmentally friendly and high-performance alternatives to traditional propellants. The current efforts in green space propulsion generally focus on two main objectives, which are the replacement of hazardous propellants and the identification of propellants with less harmful environmental impact [75]. Notably, the ongoing trend of replacing ammonium perchlorate (AP) in green propellants revolves around. AP is the most common inorganic oxidizer, responsible for releasing oxygen to facilitate the combustion of fuel in propellants. Nonetheless, a primary limitation of AP in propellants systems arises from the production of chlorinated compounds during its combustion, leading to environmental concerns such as acid rain and ozone depletion in the stratosphere [76]. Ammonium nitrate (AN) serves as an alternative to AP, but it is less energetic and has a slower burn rate [77]. Additionally, the use of dual-oxidizer systems, such as AN with ammonium dinitramide (ADN), is highlighted as having potential advantages, including enhanced specific impulse and lower hydrogen chloride (HCl) production [78], thereby reduced or low toxicity. Larsson and Wingborg have reported that ADN, seems promising as a green substitute for AP [79]. Another research has focused on hydrazinium nitroformate (HNF) and its derivatives as promising candidates in the necessity for superior and environmentally friendly oxidizers for future solid rocket propellant formulations [18]. HNF exhibit a superior heat of formation compared to AP, enabling them to potentially offer propellants with higher performance levels despite having a lower oxygen balance. Additionally, they undergo highly exothermic combustion reactions near the surface, unlike nitramines, which results in efficient heat feedback to the deflagrating surface and enhances burning rate. The utilization of green oxidizers such as ADN and HNF combined with innovative energetic binders like glycidyl azide polymer (GAP), polyglycidyl nitrate (PGN), polynitromethyloxetane (PLN), and 3,3-bis(azydomethyl) oxetane (BAMO), holds promise for achieving improved propellant performance in terms of specific impulse. Moreover, this combination has the potential to generate environmentally acceptable combustion products. Their work contributes to the development of environmentally sustainable propellants that offer enhanced performance characteristics.

In addition, natural or green stabilizers which are usually readily available have gained attention for enhancing propellant stability while reducing toxicity and environmental impact compared to conventional stabilizers [28,80]. Conventional stabilizers, such as aniline derivatives, can form toxic and carcinogenic compounds during storage. In contrast, natural stabilizers like curcumin and guaiacol offer effective stabilization with lower toxicity risks [15,17]. Studies have shown that natural stabilizers, including Organosolv lignins and Kraft lignin provide superior chemical stability in NC-based propellants [25,26]. These compounds trap decomposition products, delaying further degradation and improving thermal stability, making propellants more resistant to decomposition at elevated temperatures. Additionally, natural stabilizers such as zeolites are compatible with various propellant components, ensuring they do not adversely affect performance [81]. The use of natural or green stabilizers addresses environmental and health concerns associated with conventional stabilizers, offering a promising alternative for enhancing the stability of solid propellants by reducing toxicity, improving chemical and thermal stability, and providing environmental benefits.

## 3 Nitrate ester and decomposition mechanism

Nitrate ester are high-energy nitro functional groups in energetic materials that are contain O-NO_2_ chemicals bonds in their structures. The thermal decomposition of nitrate ester involves the breaking of O−NO_2_ bonds, resulting in the release of NO_2_ gas. Subsequently, the remaining hydrocarbon structures also decomposed to yield aldehydes and other fuel fragments, which undergo oxidation facilitated by NO_2_ [82]. This oxidation process is remarkably exothermic, leading to the generation of high-temperature combustion products. NC is the one of the NE is a vital component in propellants, possesses unique physicochemical properties, such as solubility in different solvents and viscosity, which influence the nitrogen content and the applications of NC. The synthesis of NC involves nitration of cellulose using a mixture of nitric acid with other acids (e.g: phosphoric, sulfuric acid, acetic acid) and water, leading to the replacement of hydroxyl groups with nitro groups [83]. By controlling the mixed acid concentration and ratios, various degree of nitration in NC can be achieved. The degree of nitration involves determining the nitrogen content in the NC [84]. Different grades of NC with varying nitrogen contents are used in various applications.

Typically, long-distance shooting with large caliber cannons, which require higher bullet speeds and consequently more energetic propellants, necessitates the use of DB propellants. NG is commonly used in DB propellants, often combined with NC and other energetic materials. DB propellants offer several advantages over SB propellants; for instance, exhibit greater performance variance. However, DB formulations such as NC + NG may experience exudation during storage, where NG tends to migrate out of the composition, leading to inconsistencies in propellant strength and reduced firing accuracy [85]. NG is synthesized using mixed acid at low temperatures and serves as a plasticizer for NC propellant, enhancing its flexibility and improving the mechanical properties contributing to the structural integrity and performance of the propellant. Owing to the low melting point at 287 K, the amount of NG in propellants needs to be restricted to avoid excessive degradation of grain strength [86]. Like NC, NG is composed of a hydrocarbon structure with -O−NO_2_ bonds as oxidizer fragments. The thermal decomposition of NG is fundamentally the same as that of NC, producing NO_2_ as an oxidizer and aldehydes as fuel components.

Nitroguanidine is commonly found in triple-base gun propellants, existing not as a solubilized component in NC/NG mixtures but as a finely powdered inclusion within these mixtures. Incorporating nitroguanidine particles into a double-base propellant forms a composite propellant termed a triple-base propellant, as used in guns. The primary functions of nitroguanidine are two-fold: moderating the propellant's temperature to minimize gun barrel erosion and suppressing the fireball flash at the muzzle [82]. While NG is highly sensitive to impact, approximately an order of magnitude more than NC, nitroguanidine is remarkably insensitive, demonstrated by its ability to withstand maximum settings on impact sensitivity machines. Nitroguanidine's density is low and its heat of explosion is also comparatively low. However, the mass of its combustion products is low because of the high mass fraction of hydrogen contained within the molecule. Incorporating nitroguanidine particles into a double-base propellant forms a composite propellant termed a triple-base propellant, as used in guns [57]. Pentaerythritol tetranitrate (PETN) shares similarities with NC and NG as a nitrate ester. Despite being among the most potent energetic materials utilized in explosives, PETN does not yield excess oxidizer fragments upon decomposition [82]. Consequently, PETN is not employed as an oxidizer in propellants. Table 3 shows the heat of formation, *ΔH*_*form*_, heat of explosion, *H*_exp_ and nitrogen content, *N* for NEs energetic materials that commonly used as major components of propellants.

**Table 3:** The heat of formation, *ΔH*_*form*_, heat of explosion, *H*_exp_ and nitrogen content, *N* for NEs energetic materials [82].

Material	Chemical Formula	Δ*H*_*form*_ (MJkg^−1^)	*H*_exp_ (MJkg^−1^)	*N* (%)
NC	(C_6_H_7_N_3_O_11_)n	−2.60	4.13	14.14
NG	(ONO_2_)_3_(CH_2_)_2_CH	−1.70	6.32	18.50
Nitroguanidine	CH_4_N_4_O_2_	−0.77	2.88	53.83
PETN	(ONO_2_)_4_(CH_2_)_4_C	−1.59	5.90	17.72
EGDN	(CH_2_)_6_O_2_(ONO_2_)_2_	−2.53	3.14	11.67

In large-scale production, it is crucial to guarantee the absence of acid in the NEs material to ensure its prolonged stability. The accumulation of acid in NEs mixtures is widely recognized for triggering exothermic autocatalytic decomposition, posing a significant risk of severe explosions. In addition, it has been reported that NE-based propellants experience degradation even in normal conditions due to the thermal degradation [6,87]. The physiochemical and ballistic characteristics of NE-based propellants can undergo changes over time, occurring during usage and storage. Consequently, a decrease in stability and a loss of performance may occur, resulting in various drawbacks. Therefore, the research focus has been notably directed towards investigating the stability of NE based-propellant. Numerous researchers have investigated the stabilizing effect of various types of stabilizers added to propellants [[88], [89], [90]]. Different types of stabilizers are used for different propellant formulations and applications, depending on factors such as the specific chemical composition of propellant, the required performance characteristics and the expected storage and usage conditions [91]. The choice of stabilizer significantly influences the overall characteristics and functionality of the propellant, making it a critical component in propellant development and application [6,92].

**Fig. 4:**
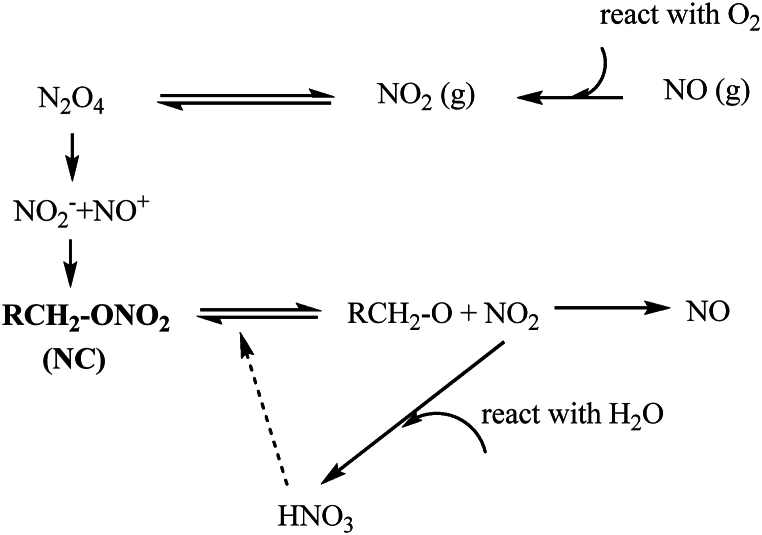
Mechanism of nitrate ester decomposition.

The NE-based propellant tends to undergo decomposition (Fig. 4) over time, decompose due to the weakness of their O-N bond (155 kJ mol^−1^) [93,94] and releasing nitrogen oxides as the most reactive part of the decomposition products. These nitrogen oxides are responsible for the autocatalytic decomposition thereby reducing the service lifetime and generating heat [40,87]. This can lead to self-ignition due to accelerated decomposition caused by autocatalysis and/or self-heating [28,95]. To address this issue, stabilizers are incorporated into propellant formulations, react with nitrogen oxides to prevent autocatalytic decomposition that affects to the changes in chemical and physical properties, leading to the deterioration of performance, stability and safety of propellants. Natural degradation involves the breakdown of chemical constituents within the propellant due to thermal effects, chemical reactions or prolonged storage [96]. NE-based propellants can be decomposed by two main processes which are thermolysis and hydrolysis, as described by Chin et al. [97]. In the absence of a stabilizing agent, the propellant inherent instability that can lead to the degradation of NC-based propellants, resulting in autocatalytic decomposition and spontaneous ignition. In general, the O-NO_2_ bond in the nitrate compound is broken at the beginning termed as thermolysis and leads to the formation of alkoxyl radical and nitrogen oxide as shown in equation (1).

(1) RCH_2_ONO_2_ → RCH_2_O^●^ + ^●^NO_2_

The reactive species that are form catalyzes the decomposition of nearby nitrate ester molecules, allowing subsequent decomposition reactions. As a result, various of decomposition products forms [98] as in equation (2). Breakage of an N−N or O−O bond generates heat with the formation of CO_2_ or N_2_ as a reaction product.

(2) RCH_2_O^●^ + ^●^NO_2_ →R’ONO_2_ + NO + N_2_O + N_2_ + N_2_O_4_ + NO_2_ + CO_2_ + CO

The release of more NO_2_, resulting in the formation of a cyclical pathway that will accelerate the decomposition of NC and uncontrolled self-heating [99]. On the other hand, the presence of water in the surrounding environment or on the NE compund promotes the hydrolysis reaction of the O-NO_2_ bond to form nitric acid (HNO_3_) as shown in equation (3). HNO_3_ react as strong oxidizers [62], engages in electrophilic reaction, breaking more bonds in the polymer and generating more NO_2_ which enhances the catalytic action reaction.

(3) RONO_2_ + H_2_O → ROH + HNO_3_

These reactions can lead to accumulation of heat in the propellant system and when temperature is increased above 180 °C, the propellant can ignite spontaneously without an external spark [100]. Moreover, the uncontrolled production of NO_2_ leads to an autocatalytic reaction that may resulting in a dangerous situation. The reaction proceeds with a lot of NO_2_ forming an organic free radical which is much more reactive than the NO, and carbon-carbon bond cleavage can occur [101]. The major products from the reaction are aldehydes, with evolution of NO_2_ gas, resulting in alcohols will continue to accelerate the decomposition of NC. Therefore, the main reason for the low thermal stability of NE is due to the formation of nitrogen oxide radicals and acid gases generated during the thermal decomposition of nitrate bonds [102]. Therefore, the physicochemical properties of the propellants change as NE decomposes over time.

The autocatalytic reaction can be slowed down by adding stabilizers in propellant formulations [103,104]. Stabilizers works by binding the nitrogen oxides, eliminating water or neutralizing acids in the system and avoiding them from reacting with NC or NG [88].

There are several factors that can affect the chemical properties and stability performances of the propellant which are: (i) Formulation and compatibility of the propellant [54,105,106]: The selection of the chemical substance and the ratio of formulations propellant play a significant role in the propellant's stability. The content of stabilizers and the compatibility within energetic materials, oxidizer, binder and other additives influenced the stability throughout the life cycle of the propellant. The vacuum stability test (VST) is a commonly used method to determine the compatibility of materials in propellant formulation [107]. A study has been conducted by Rodrigues et al. (2022) focused on the replacement of 1 mass-% traditional stabilizers with 1 mass-% guaiacol in NC-based propellants [108]. The study evaluated the homogeneity, stability, and compatibility of the propellant samples and the results showed that the guaiacol stabilized samples were stable and did not degrade for more than 3 days when subjected to a constant temperature of 100 °C.

(ii) Temperatures, moisture and storage condition [109]: The decomposition process in propellant occurs rapidly at higher temperatures. At elevated temperatures above 170–180 °C, NE become highly explosive and decompose rapidly without external spark. Therefore, storage and operating temperatures conditions must be controlled to minimize the negative effects on the stability of the propellant. Propellant is typically stored at temperature below 40 °C or 50 °C as stated in Ref. [100], to ensure the propellant's longevity and performance with the recommended temperature range of 15 °C–30 °C [110]. This condition providing a secure environment for storage operation. However, study reported by Kumar et al. (2021) found that there is a loss of stabilizer with time in ambient conditions during ageing, which can affect the stability of the propellant [111]. This suggests that even under normal storage temperatures, the stability of the propellant may be compromised over time. Other than that, the presence of moisture also can cause the degradation of propellant by promoting hydrolysis on the nitrate groups. Moisture can lead to changes in propellant properties such as altered the burn rates and reduced stability of propellant. The moisture control is essential to ensure prolong propellant stability. For instance, the effect of relative humidity and absorbed water on the ethyl centralite stabilizer consumption in NC-based propellants was investigated by Teixeira et al., 2023 and suggesting that humidity levels during storage can influence the stability of the propellant [112].
(iii) Oxygen Exposure [54]: The effect of oxygen exposure on the propellants can lead to the formation of degradation products such as nitrogen oxides, which can degrade the propellant components and reduce in performance and stability. In addition, Reshmi et al. (2014) focused on the effect of oxygen exposure on the thermal decomposition behavior of propellants and the study provides further evidence that oxygen exposure has negative effects on propellant stability [113]. The oxygen content can significantly influence the combustion behavior and burning characteristics of the propellant. Overall, oxygen exposure affected in the combustion, energy release, and degradation of propellants, making it a critical factor to consider in propellant design and application. Therefore, propellant is often stored in sealed container or under an inert atmosphere to minimize the oxygen exposure and mitigate these negative effects.

## 4 Research development of Stabilizer in nitrate ester-based propellants

**Fig. 5:**
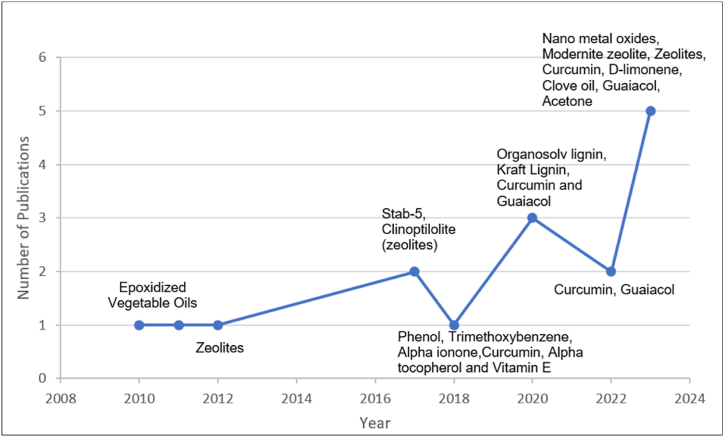
Research development of green stabilizer of NE-based propellants. (For interpretation of the references to colour in this figure legend, the reader is referred to the Web version of this article.)

Recent years have witnessed significant interest in the research progress of stabilizers for NE-based propellant [6,63,87,114]. Stabilizers play a critical role in propellants by absorbing nitrogen oxides (NOx) and preventing the auto-catalytic decomposition of NE. This function extends the service life of NE-based propellants, ensuring their stability and sustained performance over time. The stability assessment of stabilized NE-based propellants has been a central focus, employing analytical methods for ensuring their safe and effective use. Tao et al. delved into the aging performance of Nitrate Ester Plasticized Polyether (NEPE) propellants, assessing the stabilizer content of these propellants via Fourier Transform Infrared Spectroscopy (FTIR) to analyze their aging behavior [115]. Meanwhile, Sun et al. investigated the interaction mechanism of aromatic amines stabilizer with NE propellants [116]. Notably, the commonly used stabilizers can form potentially toxic and/or carcinogenic nitrosamine derivatives during prolonged storage of propellants and there is an effort to discover alternative stabilizers without any amine moiety to avoid nitrosamine formation. Understanding the interaction between stabilizers and NE is pivotal in influencing the aging and thermal decomposition of propellants, aiding in the prediction of their operational life-time. Additionally, there is a pressing need for non-toxic stabilizers to stabilize NE-based propellants, without giving rise to carcinogenic or mutagenic substances [28]. Thus, this section highlights ongoing research progress on environmentally friendly stabilizers, known as green stabilizer. Table 4 presents detailed data, while Fig. 5 features a curve chart illustrating the development process of green stabilizers for NE-based propellants.

**Table 4:** Research studies on green stabilizer for nitrate ester propellants.

Year	Types of propellant	Stabilizer	Main Finding	Reference
2023	SB-NC propellant	Curcumin, D-limonene, Clove oil, Guiaiacol, Acetone	The incorporation of essential oils in conjunction with phenolic natural stabilizers enhances stability properties not merely through a cumulative effect, but synergistically.	[126]
2023	SB-NC propellant	Zeolites: Mordenite, Sodium (MOR), β- ammonium (BEA), ZSM-5, ammonium (MFI), Faujasite Y (FAU)	Various zeolites demonstrated promising efficiency in stabilizing NC. Additionally, these zeolites exhibited good compatibility with NC.	[81]
2023	SB-NC propellant	Mordenite zeolite	Modernite zeolite porous material exhibit effective stabilization of NC, thus can replaced conventional DPA and reducing the release of toxic or carcinogenic byproducts.	[23]
2023	NC	Nanoparticles Barium oxide	The results of the thermal kinetic analysis and stability tests unveiled an improvement in both the thermal and chemical resistances of NC with the introduction of nano barium oxide.	[127]
2023	NC	Nano and micro-Titanium dioxide	The stability tests results showed an enhancement in the chemical stability of the pure NC by the incorporation of TiO_2_ nanoparticles. Furthermore, the nanoscale metal oxides exhibited a superior stabilizing effect compared to their micro sized metal oxides.	[128]
2022	SB-NC propellant	Curcumin	Curcumin has the potential to serve as a substitute for DPA in stabilizing SB propellants.	[16]
2022	SB-NC propellant	2-methoxy phenol, known as guaiacol	Guaiacol proves to be a highly effective and efficient alternative to DPA as a propellant stabilizer for single-base NC propellants, thereby making them more environmentally friendly.	[17]
2020	SB-NC propellant	Curcumin and guaiacol	The curcumin stabilizer possesses no toxicity, whereas guaiacol may have carcinogenic and mutagenic properties. Results from the heat-flux microcalorimeter (HFC) indicate that the new propellants are more stable compared to those using traditional stabilizers.	[15]
2020	NC sample	Softwood and hardwood Kraft lignins (KL) obtained from Aleppo pine (AP) and Eucalyptus globulus (EG)	Both green organic compounds KL (AP) and KL (EG) have the potential to replace conventional stabilizers due to their availability, sustainability, renewability, and operational readiness.	[26]
2020	SB-NC propellant	Organosolv lignin (OL), obtained from Aleppo pine (AP) and Eucalyptus globulus (EG)	The results obtained reveal that both lignin OL (AP) and OL (EG) can efficiently substitute conventional stabilizers due to their renewability, availability, sustainability, and operational readiness.	[25]
2018	DB powder	2,3,5-Trimethylphenol,1,2,3 trimethoxybenzene, Alpha ionone, Curcumin, Alpha tocopherol, Vitamin E	Stability assessments were effectively conducted for propellants containing up to 40 % of NGL and approximately 1 % of each new "green" stabilizer. The resulting daughter products from these stabilizers will identified in future study.	[28]
2017	DB propellant	Zeolite stabilizers (nano- and micro-clinoptilolite)	Nano-clinoptilolite can be regarded as an effective stabilizer for DB propellants. The percentage 4 % w/w of nano-clinoptilolite exhibited superior stability performances compared to different percentages of nano-clinoptilolite	[129]
2017	SB-NC propellants DB rocket propellants	bis(2,6-dimethoxyphenyl)triethyleneglycol (Stab-5)	Stab-5, has demonstrated to be high effective stabilizer for NC based gun and rocket propellants with expected lifetime that exceeding 100 years	[22]
2012	DB propellants	Nano-clinoptilolite	This study has revealed that samples containing the new stabilizer at percentages of 3.0 %, 3.5 %, and 4.0 % exhibit superior stability effects for DB propellants compared to the conventional stabilizer. Additionally, the effectiveness of each inorganic stabilizer in stabilizing DB propellants is justified and linked to its structure.	[130]
2011	DB propellant (NC + NG + stabilizer)	Epoxidized oils: linseed oil, epoxidized soybean oil, and mixture of fatty acids	The chemical structure of these novel stabilizers and their decomposition byproducts, prevents the formation of toxic N-nitrosamine.	[118]
2010	DB propellant (NC + NG + stabilizer)	Epoxidized oils: soybean oil, linseed oil and mixture fatty acids	The stabilizers utilized in the research are epoxidized vegetable oils which, unlike conventional stabilizers meet the current standards for human and environmental toxicity.	[131]

∗ SB is single-base, NC is nitrocellulose, DB is double-base, NG is nitroglycerine.

### 4.1 Conventional Stabilizer

**Fig. 6:**
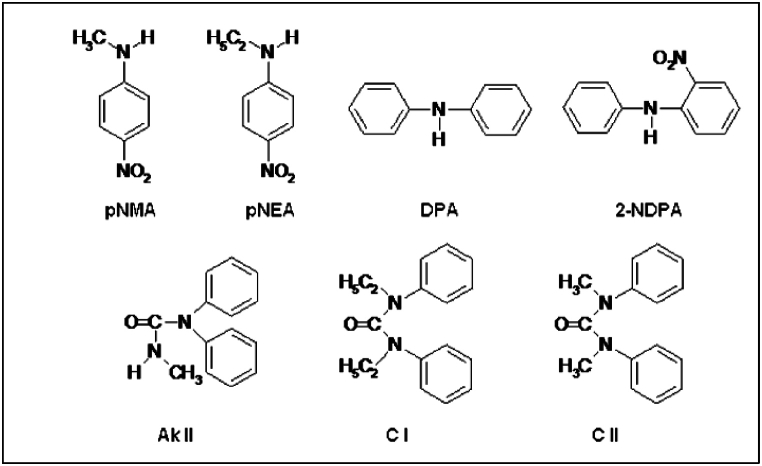
Chemical structure of conventional stabilizers.

Stabilizers commonly used in propellant formulation can be categorized into two groups: aromatic amines-based stabilizer and urea-based stabilizer (Fig. 6). DPA is one of the example of aromatic amines that extensively used in SB propellants or multi-based propellants. It has the function to absorb nitogen oxides, binding degradation products and convert them to various C-nitro and N-nitroso derivatives, thus extending storage times [117]. Over time, DPA degrades into consecutive products such as 2-nitro-DPA (2-NDPA) and 4-nitro-DPA (4-NDPA) and notably, the chemical structure of DPA and their decomposition products can lead to the formations of toxic N-nitrosamines and carcinogenic of N-nitrosodiphenylamines. Frys et al. revealed that freshly produced DPA-stabilized propellant, typically contain concentrations of N-nitrosamines, ranging from 0.1 % to 0.5 % or greater [118]. In aged DB propellants, the N-nitrosamines content increased up to 1 % in DPA-stabilized propellants. Another study by Xie et al. emphasized the significance of accurately monitoring DPA content in propellants for storage, transportation, and utilization, as the chemical stability of aged SB propellant is closely dependent on the remaining DPA content [119]. Additionally, research by Heil et al. found that propellant samples aged over 35 years, stored at 35 °C or 40°, still contain significant amount of 2-nitro-DPA and 4-nitro-DPA, highlighting that DPA is enduring activity as a stabilizer even after prolonged exposure at slightly elevated temperatures [120]. DPA is the most critical stabilizer need to be replaced since DPA is toxic by itself and always contains extremely carcinogenic impurity and generates large amount of N-nitrosodiphenylamine (more than 0.1 % in freshly produced propellant).

N-methyl-nitroaniline, (MNA) is another widely utilized aromatic amines-based stabilizer, recognized for its efficiency as a stabilizing agent in propellant compositions [98,121]. The function of MNA as a stabilizer lies in its ability to neutralize the free radical nitrogen oxides (NOx) released from the initial decomposition of NE, thus preventing the auto-catalytic reaction facilitated by its low activation energy. Moreover, Gu et al. reported that the presence of MNA helps to maintain the tensile strength and cross-linked density of the propellant, even as NE decompose, resulting in a more stable and reliable propellant over time [122]. MNA is depleted during the process and as MNA stabilizer is consumed, the decomposition of NE accelerates gradually. While possessing a notable capacity for retaining nitrogen oxides, MNA exhibits less compatibility with NC and NG, leading to potential crystallization issues and thereby limiting its application in solid propellant formulations [121].

Urea-based stabilizers like centralite (CI and CII) and akardite (AkI and AkII) are widely employed in propellants for their ability to enhance thermal stability and prolong shelf life [100,123]. Centralite stabilizer reacts with volatile NC decomposition products, yielding various centralite derivatives such as 2-nitroethyl centralite, 4-nitroethyl centralite, 2,4-dinitroethyl centralite, 4,4-dinitroethyl centralite, N-nitroso-ethylaniline, and N-nitroso-4-nitroethylaniline [124]. Additionally, ethyl centralite serves a dual purpose as both a stabilizer and a plasticizer for NC. However, the low solubility and diffusivity of gaseous byproducts generated during the stabilization process render centralite unsuitable for use in propellant grains with substantial web thickness. Nitrosation and nitration reactions of centralite result in the emission of carbon dioxide (CO_2_), which, when released during the reaction, may contribute to cracking observed in propellants with large web thickness [88]. Klerk have reported that, centralite (ethyl and methyl) exhibits greater stability effectiveness than DPA in gun propellants with high NG content in DB-propellant [98]. Furthermore, ethyl and methyl centralite demonstrate better compatibility with NG over DPA. Nevertheless, a study by Fryš et al. (2011) indicated that N-nitroso alkylanilines derived from centralite exhibit higher toxicity compared to N-nitroso-DPA produced from DPA or 2-NDPA [20]. Akardite is another urea-based stabilizer that can be used in DB propellants. The stability of akardite II (AK-II) is greater than DPA as well as centralite I (C-I) [123]. Additionally, the freshly produced AK-II stabilized propellant is less toxic compared to the DPA stabilized propellant [20]. It has been observed that freshly produced propellants mixed with AK-II stabilizer usually have N-nitrosamine concentrations ranging from 0.01 % to 0.1 %. On the other hand, freshly produced propellants stabilized with DPA contain higher concentrations of N-nitrosamines (primarily N-NO-DPA), typically ranging from 0.1 to 0.5 % or even higher.

Bellamy et al. investigates the effectiveness of various dual stabilizer systems in prolonging the shelf life of cast DB rocket propellants [125]. Typically, stabilizer systems of this nature involve two secondary amines and it is commonly assumed that the more reactive amine fast interacts with propellant decomposition products in the initial aging phases, while the second amine exhibits a slower and independent reaction, particularly after the initial stabilizer has been fully utilized. The study examines the behavior of various stabilizers, including para-nitro-N-methylaniline (pNMA), structurally related secondary amines like p-nitro-N-ethylaniline (pNEA) and 4-nitrodiphenylamine (4NDPA), along with resorcinol, in combination with 2-nitrodiphenylamine (2NDPA) in solutions containing nitroglycerin (NG). Overall, the study highlights the importance of a balanced dual stabilizer system in reducing NE decomposition and stabilizer depletion rates. It suggests that combinations of stabilizers beyond pNMA/2NDPA could potentially offer improved storage properties for CDB propellants. Additionally, the findings emphasize the need to consider both kinetically and thermodynamically controlled stabilizer reactions for designing effective stabilizer systems.

Overall, conventional stabilizers for NE-based propellants are effective but pose serious environmental and health risks due to their toxic and carcinogenic by-products. This has led to a growing interest in natural or green stabilizers, which offer similar or better effectiveness without the associated hazards. The shift towards these safer alternatives is crucial for both human health and environmental sustainability.

### 4.2 Green Stabilizer

a) Natural substance (Epoxidized oils, Curcumin, Guaiacol, Essential oils, Vitamin E, Wood lignin)

Epoxidized oils refer to a type of oil that has undergone a chemical process called epoxidation, wherein double bonds in the oil's unsaturated fatty acids are reacted with a peracid or peroxide to form epoxide groups [132]. This process results in the conversion of the double bonds into epoxide rings, which can impart various desirable properties to the oil, such as increased stability, improved resistance to heat and oxidation, and enhanced flexibility. Frys et al. have innovatively introduced epoxidized oils such as soybean oil, linseed oil, and a mixture of fatty acids, formulated specifically as stabilizers in DB powder propellants. These stabilizers are incorporated into propellant compositions ranging from 0.9 % to 2.0 % w/w and tailored to accommodate diverse shapes of the final propellant grain [118]. These stabilizers were tested using microcalorimetry and conventional stability tests; such as Bergmann-Junk test, methyl-violet test, weight loss test and vacuum stability test. Microcalorimetry test are done at 89 °C are based on the standard STANAG 4582 in which this technique necessitates a maximum heat flow of 314 mW g^−1^ within the initial 3.83 days of testing. This requirement is securely satisfied by all stabilized samples. Consequently, propellant powders stabilized with these epoxidized oils can be stated to maintain chemical stability for at least 10 years at a storage temperature of 25 °C, as their maximum heat flow rates are all below the acceptable threshold of 314 mWg^−1^. The weight loss test for DB propellants requires that the weight loss should not exceed 3 % within a 20-day period of testing at 89 °C. In this case, all oil-stabilized samples meet this standard.

The results indicated that epoxidized oil stabilizers effectively stabilized the propellants without enabling the formation of toxic N-nitrosamines. Furthermore, all samples passed the conventional stability tests demonstrating the effectiveness of the new stabilizers. The toxicological properties of these epoxidized oils are presented in Table 5. Overall, these findings suggest that epoxidized oils, including soybean oil, linseed oil, and a mixture of fatty acids, are safe for use and do not pose significant health risks under the tested conditions. The main finding of this study lies in the successful development and application of new nontoxic stabilizers, specifically epoxidized oils-based, for use in smokeless powders of DB propellants. These stabilizers effectively prevented the decomposition of NE-based propellants, thereby improving the stability during storage. Importantly, these new stabilizers do not produce toxic N-nitrosamines, distinguishing them from conventional stabilizers.

**Table 5:** Toxicological properties of epoxidized oils.

Composition	Acute Toxicity LD_50_ (gkg^−1^)	Oral Dermal	Chronic Toxicity	Genotoxicity, Ames Test	Carcinogenicity, Mutagenic, and Teratogenic Data
Epoxidized Soybean oil	21–40	>20	Negative	Negative	Negative
Epoxidized Linseed Oil	>20	>20	Negative	Negative	Negative
Epoxidized Mixture Fatty Acids, C14–22, 2-Ethylhexylesters	38.8	>20	Negative	Negative	Negative

The study on the same epoxidized oils as stabilizer is expanded in another report, focusing on both qualitative and quantitative analysis to evaluate the effectiveness of stabilizers within propellants [131]. A gravimetric determination method is employed to quantify the presence of oils as stabilizers in the powder. This involves dissolving the sample in 70 % methanol, separating out nitroglycerin, filtering the solution, and transferring the oil component to a flask using diethylether. After evaporation and drying, the weight of the oil is measured to determine its content. Simultaneously, qualitative analyses are conducted using infrared spectrometry to identify the presence of oil stabilizers in the powder. This process includes measuring the infrared spectra of the samples and comparing them with standard oil spectra. To isolate the oil spectra from interference by nitroglycerin, additional steps are taken including the addition of methanol to the extract and dissolution of residual material in acetone, then the resulting solution is then measured. These methods provide valuable insights into the composition and stability of smokeless powders, which are essential for their safe and effective use. Additionally, the content of stabilizers after artificial ageing of propellants are determined to further assess their performance.

In another report, five new green molecules have been discovered to effectively stabilize nitrate ester, showing comparable or superior performance to industrial conventional stabilizers [28]. These molecules were selected from commercially available options, such as curcumin (can be extracted from the roots of turmeric) and vitamin E. Stability tests were conducted at varying temperatures using propellants containing up to 40 % of NGL (nitroglycerin) and approximately 1 % of each new stabilizer. Ongoing efforts involve identifying the daughter products resulting from these stabilizers and evaluating their toxicity. Additionally, the stability of these daughter products in propellant powder will be determined, followed by kinetic analyses to assess stability at lower temperatures, including room temperature. Dejeaifve et al. expanded upon this study by providing validated lower temperature shelf-life predictions for propellants incorporating these novel stabilizers. A minimum shelf-life has been established for the new propellant solutions containing up to 40 % NG, demonstrating their efficiency comparable to or better than current industry standards. These green solutions exhibit minimal dependency on the NG levels in the propellant, which offers an additional advantage. They have manufactured green propellants on an industrial scale, facilitating comprehensive characterization and validation according to standardization agreements like AOP7, that can replace hazardous stabilizers like Ak II and DPA. In a separate study, the same research group investigated the use of tocopherol stabilizers, commonly present in vegetable oils, asserting their effectiveness in stabilizing NC-based propellants [133].

**Fig. 7:**
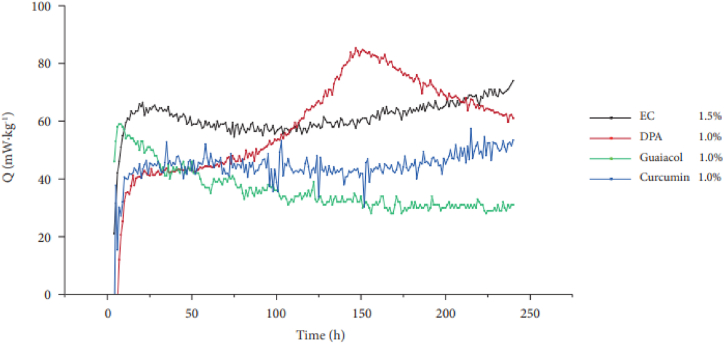
HFC curve for green and conventional stabilizer. Reprinted from the refence [15]. (For interpretation of the references to colour in this figure legend, the reader is referred to the Web version of this article.)

Rodrigues et al. explored curcumin and guaiacol were explored as natural substitutes for traditional stabilizers on replacing traditional carcinogenic and toxic stabilizers in NC-based propellants while maintaining stabilization efficiency and shelf-life [15]. Curcumin and guaiacol were explored as natural substitutes for traditional stabilizers. Chemical stability evaluations using a HFC indicated that propellants with these new stabilizers are more stable than those with traditional stabilizers like DPA and EC as shown in Fig. 7. Computational toxicity analyses showed curcumin to be non-toxic, while guaiacol exhibited only potential carcinogenic and mutagenic properties highlighted in Table 6. The conclusion highlights successful incorporation of curcumin and guaiacol into NC-based propellants, with curcumin showing promise as a non-toxic alternative. Further characterization involving stability tests, degradation assessments, and investigation of by-products during NC degradation is ongoing, particularly to assess the toxicity of these by-products. These compounds have the capability to capture alkoxy radicals produced during the degradation of NC, subsequently forming new alkoxy radicals that are relatively stable. According to Rodrigues et al. [17], guaiacol was selected because amine or amide groups are not present in their structure, reducing the tendency of generating carcinogenic, mutagenic, or reproductive toxic derivatives during stabilization. Additionally, it offers the potential to enhance the shelf life of propellants, aiming for formulations lasting 10–15 years, surpassing conventional ammunition. This could result in cost savings by reducing replacement rates and minimizing the need for extensive testing and disposal operations.

**Table 6:** Prediction of carcinogenic and mutagenic for conventional and green stabilizers using various software tools.

Software	VEGA	Toxtree	LAZAR	TEST
DPA	Noncarcinogenic	Noncarcinogenic	Carcinogenic	Nonmutagenic
	Nonmutagenic	Mutagenic	Mutagenic	
EC	Noncarcinogenic	Noncarcinogenic	Noncarcinogenic	Nonmutagenic
	Nonmutagenic	Nonmutagenic	Nonmutagenic	
Curcumin	Noncarcinogenic	Noncarcinogenic	Carcinogenic	Nonmutagenic
	Nonmutagenic	Nonmutagenic	Nonmutagenic	
Guaiacol	Carcinogenic	Noncarcinogenic	Noncarcinogenic	Nonmutagenic
	Nonmutagenic	Nonmutagenic	Mutagenic	

Guaiacol and curcumin are natural occurring compound that are referred as green stabilizer due to none of nitrosamine by-product formation. However, the aromatic rings on these molecules have their acidity augmented when capturing the NOx groups and, paradoxically, increasing hydrolysis rate. In order to find a solution for the problem, Silva et al. evaluated the use of essential oils containing terpenes and/or phenolic compounds to introduce a buffering capacity [126]. Several propellant formulations incorporating these compounds were subjected to traditional stability tests and the results presented in Table 7. Results suggest that essential oils not only enhance stability properties individually but also synergistically. Limonene and clove oil, specifically, were employed in this study to mitigate the catalytic effect of increased acidity without compromising stabilization. The findings indicate a synergistic enhancement of stabilizing properties. From this study, they found that, the use of guaiacol and curcumin as stabilizers increases acidity, but this can be counteracted by essential oils like clove oil and limonene. Results showed that curcumin and guaiacol, along with combinations of these compounds with clove oil and limonene, effectively stabilized an unstable NC. Further tests, including pressure vacuum stability test and heat flow microcalorimetry, confirmed that formulations containing guaiacol and curcumin with limonene stabilized the propellant according to standards. Clove oil, due to its mixture of phenolic compounds and terpenes, enhanced stabilization when mixed with guaiacol. Curcumin alone performed better than reference stabilizers, but not when combined with limonene. Guaiacol and its combinations with eugenol and limonene have been fabricated for industrial batches and demonstarted satisfactory ballistic results. Overall, the findings suggest that the use of essential oils alongside phenolic natural stabilizers enhances stability properties synergistically.

**Table 7:** Results of traditional stability test.

Sample	Bergmann-Junk (132 °C)/VNO (mL)	Storage (100 °C)	German (134.5 °C)
Pure NC	Unstable/3.70	Unstable	Unstable
Curcumin	Stable/0.74	Stable	Stable
EC	Stable/0.94	Stable	Stable
Guaiacol	Stable/0.87	Stable	Stable
DPA	Stable/0.52	Stable	Stable
Guaiacol (0.8 wt%) + Clove oil (0.2 wt%)	Stable/0.83	Stable	Stable
Curcumin (0.8 wt%) + Limonene (0.2 wt%)	Stable/0.72	Stable	Stable

**Fig. 8:**
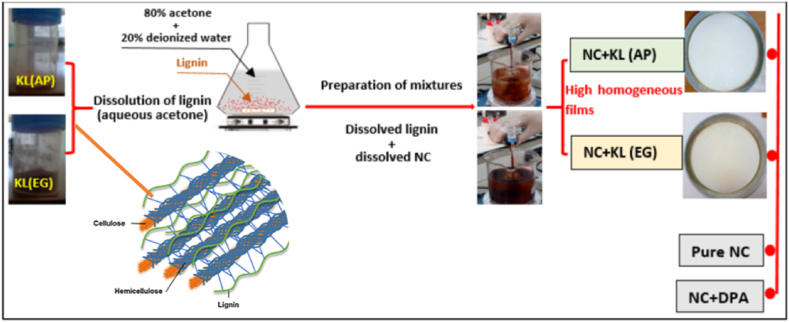
The experimental procedure for preparing NC-lignin stabilizer mixture. Reprinted with permission and modified accordingly from reference [26].

Recently, wood lignin has emerged as a potential stabilizer owing to its inherent aromatic rings, making it an excellent candidate for NO_2_ group binding. Cherif et al. have investigates the stabilizing potential of two lignins extracted from softwood (Aleppo pine, AP) and hardwood (Eucalyptus globulus, EG) using the Kraft method, in comparison to the conventional stabilizer DPA for NC [26]. In the preparation of NC-stabilizer mixture, lignin initially was dissolved in aqueous acetone under stirring until complete dissolution. The optimal solubility of lignin was achieved with 80 % acetone and the resulting solution was then combined with solubilized NC under stirring. The resulting mixture was subsequently air-dried at room temperature to obtain a homogeneous thin film. Fig. 8 illustrates the experimental procedure for preparing NC-stabilizer mixture.

**Fig. 9:**
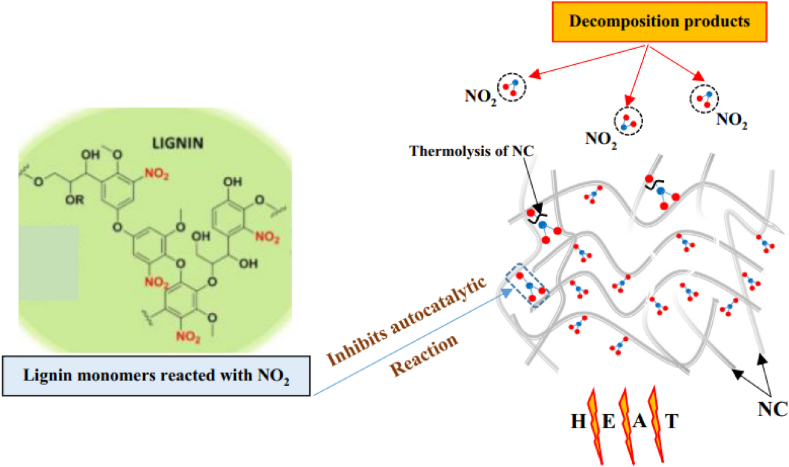
Stabilization mechanism of lignin. Reprinted from reference [25].

Various stability tests, including Bergmann & Junk test, and vacuum stability test, along with kinetic modeling using isoconversional methods, were conducted to determine kinetic parameters. Results indicate that both lignins, regardless of their botanical source, exhibit similar stabilizing effects on NC, capable of reacting with nitrous vapors and scavenging free radicals produced during NC decomposition. This suggests the potential efficiency of Kraft lignins as stabilizers for NC. The same research group has expanded their study by exploring the stabilizing potential of two organosolv lignins (OL) derived from the same of botanical sources; Aleppo pine (AP) and Eucalyptus globulus (EG) on NC. Through conventional stability tests and kinetic modeling on various sample compositions, it was found that both OL stabilizers exhibit effective stabilization. The findings suggest that these environmentally friendly and readily available substances can serve as efficient stabilizers for NC-based formulations. Moreover, the influence of the lignin source was observed, with hardwood lignin showing slightly superior stabilization compared to softwood lignin, as evidenced by TG and kinetic results. Lastly, the interaction between NC and the new stabilizers demonstrated their capability to react with nitrous vapors and scavenge free radicals, indicating the potential of organosolv lignins for NC stabilization. Based on all findings for both Kraft and Organosolv lignin, the suggested stabilization process of lignins is depicted in Fig. 9. Lignin stabilizer interacts easily with NC decomposition byproducts, forming nitro aromatic rings. Moreover, lignin demonstrates a beneficial impact on free radical scavenging activity, thereby facilitating the prevention of the autocatalytic reaction and prolonging the shelf life of NC.

b) Inorganic stabilizer (zeolites, metal oxides)

**Fig. 10:**
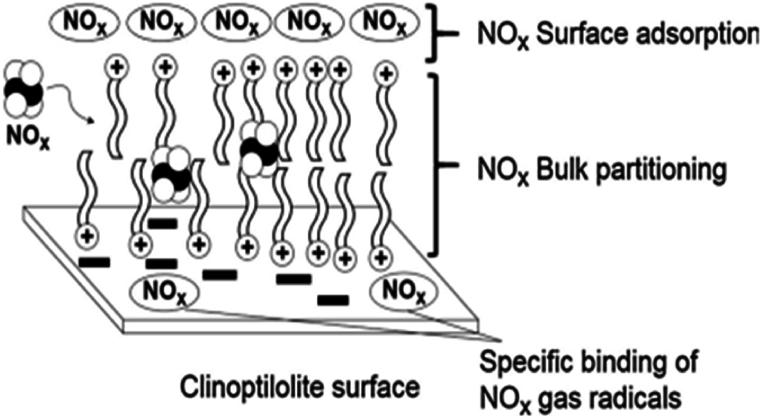
Illustration depicting the formation of a bi-layer of (NOx) in a tail-to-tail configuration on the surface of clinoptilolite. Reprinted from reference [129].

The utilization of inorganic stabilizer as replacements for conventional stabilizer is another approach to solve the problem of toxic and carcinogenic effects from nitrosamines formed by conventional stabilizer. Zayed et al. investigated the effectiveness of inorganic stabilizers, specifically on natural zeolites of clinoptilolite for DB propellants [130]. In their study, nano-scale clinoptilolite with a particle size of 30 nm with ranging from 2 % to 4 % in concentration, was evaluated to study the stabilizing effect for DB propellant using classical thermal stability tests (Bergmann–Junk and calorimetric tests) and complemented by thermal analysis measurements (TGA and DSC) and kinetic parameters calculation. As a result, clinoptilolite that composed of hydrated sodium potassium calcium aluminum silicate emerged as a promising alternative for conventional stabilizers. It was observed that the use of clinoptilolite stabilizers, could effectively address concerns associated with traditional stabilizers, particularly regarding the formation of toxic and carcinogenic nitrosamines. The stabilization mechanism of clinoptilolite involves the adsorption of nitrogen oxides in the pore openings of the clinoptilolite as well as chemi-sorption processes facilitated by its high stability even at elevated temperatures (Fig. 10). Additionally, the grain size of the inorganic stabilizer clinoptilolite was found to be a crucial factor influencing the stabilization process in which nano-clinoptilolite enhances the uniformity and homogeneity of the propellant's surface, resulting in a larger surface area that are capable of adsorbing a greater quantity of nitrogen oxides generated on its surface [129]. This result demonstrated the greater effectiveness of nano-size stabilizer compared to micro-size stabilizer in propellant stabilization reaction.

Zeolites have garnered attention as promising candidates owing to their high surface area, ion exchange capacity, and moisture-absorbing properties. Zeolites possess the ability to efficiently trap acidic species by utilizing their ion-exchange capacity and high surface area. This capability effectively inhibits these species from catalyzing additional decomposition reactions [81]. Beyond their stabilizing characteristics, zeolites offer further appeal due to their low toxicity and widespread availability. They can be readily synthesized or extracted from natural sources, and their properties can be customized to suit with specific application requirements. A study conducted by Chebbah et al. also highlights the advantages of using zeolite adsorbents as environmentally friendly and efficient alternatives to traditional stabilizers for NC [81]. This study investigates the potential of four different zeolite adsorbents as stabilizing agents for NC and comparing their performance to the commonly used stabilizer, DPA. The results indicated that the zeolite adsorbents showed promising stabilizing efficiency for NC and exhibited good compatibility with NC and were found to be more effective stabilizers than DPA. Additionally, the interaction between NC and the new stabilizers demonstrated their ability to scavenge free radicals produced during NC decomposition, further emphasizing their effectiveness as stabilizers for NC as well as environmentally friendly and efficient alternatives to traditional NC stabilizers. These findings are corroborated by a study conducted by Cherif et al. which utilized isoconversional methods to determine the kinetic properties of mordenite (MOR) zeolites, confirming the effectiveness of MOR as a stabilizing agent [23]. This suggests that such inorganic compound can be used efficiently as a stabilizer for NC-based energetic formulations. Ultimately, it can be deduced that mordenite zeolite porous material displays good stabilizing effects on NC, can effectively substituted conventional DPA, thereby reducing the release of toxic and carcinogenic derivatives.

**Fig. 11:**
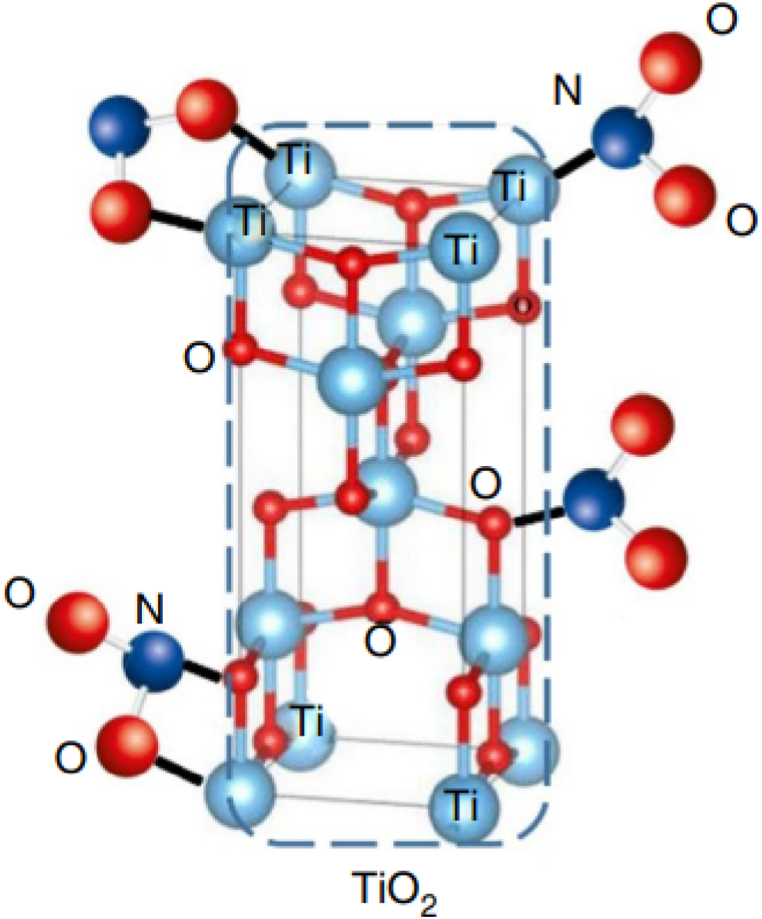
Proposed stability mechanism of nitrocellulose by titanium dioxide (TiO_2_). Reprinted with from reference [128].

The use of metal oxide nanoparticles as stabilizers in propellants has been a subject of extensive research recently. Metal oxide nanoparticles, such as titanium dioxide (TiO_2_) and barium oxide (BaO) have been investigated for their potential to enhance the stability properties of composite solid propellants [127,128]. Studies have focused on the effects of different metal oxide catalysts on the thermal decomposition of propellants, aiming to tailor their stability characteristics. Study by Touidjine et al. examined the impact of TiO_2_ nanoparticles on the chemical stability of NC through qualitative and quantitative stability tests, including methyl-violet and Abel tests, Bergmann-Junk test, vacuum stability test, and microcalorimetric measurement (HFC). A comparative analysis was conducted using commercial titanium oxide of microscopic size to highlight the influence of particle size. The obtained results showed that both nano- and microparticles of titanium dioxide enhanced the thermal stability of NC. Furthermore, the addition of TiO_2_ nanoparticles improved the chemical stability of pure NC, with nanoscale metal oxides exhibiting superior stabilizing effects compared to microsized metal oxides. The addition of TiO_2_ enhanced the chemical stability of NC during storage and increased the stabilizing effect, by reducing the heat released during thermal decomposition reactions. This reduction can occur due to the cleavage of NO_2_ groups on the activated surface of TiO_2_ or through the reaction of TiO_2_ with NO_2_ groups (Fig. 11). Analysis of the maximum heat release values and corresponding times indicates that all TiO_2_ systems remain stable for 10 years under storage conditions at 25 °C. Additionally, nanosized TiO_2_ showing superior enhancement of chemical stability compared to micro sized of TiO_2_.

In another report, Touidjine et al. investigates the influence of nanosized BaO on the thermal decomposition kinetics and chemical stability of NC. BaO nanoparticles were synthesized using the precipitation method and combined with NC. Characterization techniques including spectroscopy, microscopy, and thermal analysis were employed to assess the prepared BaO and NC-based composites. Qualitative and quantitative stability tests were conducted to evaluate the impact of BaO on the chemical stability of NC. Results indicate that the addition of nano barium oxide enhances both the thermal and chemical stabilities of NC. In addition, compatibility studies revealed good compatibility between BaO and qualitative and quantitative stability tests, with thermal analytical methods, confirmed an improvement in the chemical stability of NC by the addition of BaO. Overall, this study underscores the dual role of barium oxide in enhancing both the thermal and chemical stability of NC-based formulations, suggesting its potential as a alternative stabilizer during NC storage. These studies contribute to the development of propellant formulations with enhanced performance and tailored combustion characteristics.

c) Hindered Phenols

Various substituted phenols were suggested as new stabilizers and exhibited promising but less stabilizing effect than the conventional stabilizers [134,135]. Krumlinde et al. discovered a new type of electron rich phenols with less steric hindrance that could act both as stabilizer and plasticizing properties known as bis(2,6-dimethoxyphenyl)triethyleneglycol (referred to as Stab-5) [22]. This stabilizer is electron-rich due to the methoxy-substituents on the aromatic ring and are therefore susceptible to electrophilic substitution. This study found that, Stab-5 emerged as the most promising candidate for NC-based propellants and demonstrated excellent stabilizing properties for NC-based gun and rocket propellants, with an expected product lifetime of over 100 years [136]. During accelerated aging tests and complementary nitration reactions, the products formed from the reaction between Stab-5 and NC-based propellants were isolated and identified. These products are anticipated to have a favorable toxicity profile compared to existing stabilizers, although in-vivo and in-vitro experiments are required to confirm this. Further investigation into the toxicity and carcinogenicity of Stab-5 and its reaction products with NC-based propellants is needed. The use of stabilizers like Stab-5, which do not form N-nitrosoamines, is expected to reduce environmental impact and potentially benefit people involved in propellant handling.

d) Hindered amines- TPA

Triphenylamine (TPA) as a tertiary amine and does not have any N-H bonds, preventing the formation of carcinogenic N-nitrosamines [137]. TPA has been widely studied as a potential alternative stabilizer in propellant formulations. TPA demonstrating comparable efficiency to DPA at a 2 % concentration [6]. Research revealed that TPA depletes faster than some stabilizers but slower than DPA. TPA primarily reacts with nitrogen oxides through para-nitration reactions, potentially replacing conventional stabilizers that produce toxic derivatives. Its degradation kinetics proceed in consecutive steps without parallel reactions, with reported activation energy for consumption in the range of 129–133 kJ mol^−1^. However, TPA depletion is rapid, particularly in double-base propellants, posing challenges for stability evaluation using current methods such as the AOP 48 standard. Additionally, TPA nitro derivatives, like 4-NO_2_-TPA and 4,4′-dinitro-TPA, are found to be less effective as secondary stabilizers.

Wilker et al. provides an overview of stability tests conducted on over 30 different propellants stabilized with TPA [138]. The study delves into the decomposition mechanism of TPA, the synthesis of its subsequent products, and their effectiveness as stabilizers. Additionally, it discusses TPA's compatibility with propellant ingredients and compares its decomposition rate with that of other stabilizers. This study found TPA is deemed a suitable stabilizer for propellants. However, it is noted that TPA has two drawbacks: it is relatively quickly depleted in double-base formulations, posing challenges in meeting certain criteria, and its major subsequent products exhibit low stabilizing activity.

e) *Carbon nanomaterials-fullerene*

Carbon nanomaterials, such as fullerene derivatives, have emerged as promising stabilizers for NE-based propellants due to their excellent thermal stability and ability to scavenge free radicals. Studies have specifically highlighted the application of highly functionalized fullerene derivatives as stabilizers [32,35,139]. These derivatives have shown promise as stabilizers for NC, offering a new strategy for designing high-performance stabilizers suitable for NE-based propellant. Additionally, fullerene anisole derivatives have been synthesized and characterized for their potential application as stabilizers for NC, showcasing their role in enhancing the stability of NC-based materials [29]. The addition of fullerene-based stabilizers, aims to absorb nitrogen oxide radicals generated during the decomposition of NE in propellants. This absorption helps inhibit the autocatalytic decomposition of NE by nitrogen oxides, thereby enhancing the stability of NE-based formulations [140]. These studies reveal that fullerene derivatives exhibit superior thermal stability compared to conventional stabilizers and effectively eliminate free radicals, enhancing the stability of NE-based propellants. In another report, fullerene-malonamide derivatives stabilizers react with nitroxide radicals released during the pyrolysis of NC, preventing early decomposition and potential explosions [32].

Further research into a novel multifunctional fullerene derivative, 4,11,15,30-tetramethophenyl fullereno[1,2:2′,3']dihydrobenzofuran (C60-DBTMP), has been designed and synthesized to enhance the thermal stability of NC [35]. This derivative exhibit superior stability compared to traditional stabilizers like DPA, C2, and AKII, maintaining its performance even at high temperatures. Thermal analysis indicates that C60-DBTMP interacts with the decomposition products of NC, shifting the decomposition mechanism from a self-accelerating catalytic model to a non-autocatalytic reaction model. This shift is crucial as it helps in stabilizing the compound by inhibiting the autocatalytic decomposition process. Additionally, electron spin resonance (ESR) tests have shown that C60-DBTMP has a nitroxide radical scavenging efficiency of 73.4 %, effectively preventing the acidity changes caused by the thermal decomposition of NC. Additionally, Li et al. reported that the stability of fullerene derivatives improves with the increase in carbon chain length on the p-position of the benzene ring on C60, suggesting that molecular modifications can further enhance their stabilizing properties [32].

Overall, the incorporation of carbon nanomaterials, particularly fullerene as stabilizers in NE-based materials presents a promising path for improving the stability and performance of propellants. Fullerenes are generally considered to have low toxicity, although comprehensive toxicological studies are still ongoing. Their long-term environmental impact remains incompletely understood. Unlike conventional stabilizers, fullerene does not generate carcinogenic by-products during their stabilization. Moreover, the source materials for producing fullerene are typically derived from carbon resources.

## 5 Challenges and future direction

As the field of propellants advances towards greater environmental sustainability, researchers face several challenges in developing and implementing green stabilizers for NE-based propellants. Although striving for environmentally friendly alternatives to conventional stabilizers is commendable, it comes with its own challenges. One significant challenge lies in optimizing the performance of green stabilizers to achieve the desired level of stability and compatibility with NE-based propellants. Unlike conventional stabilizers, which have been extensively studied and refined over decades, green stabilizers may exhibit variability in their performance characteristics. This variability could stem from factors such as differences in chemical composition, synthesis methods, or purity levels among different green stabilizer materials. Furthermore, the interaction and compatibility between green stabilizers and other components of NE-based propellant formulations must be carefully considered. Propellants are complex mixtures of energetic materials, binders, plasticizers, and other additives, each playing a critical role in the overall performance and stability of the propellant. The introduction of a new stabilizer into this system may introduce unforeseen interactions that could impact the propellant's stability, shelf-life, or even its combustion behavior.

Scalability is another challenge that researchers must address when developing green stabilizers for industrial-scale propellant production. While promising results may be obtained at the laboratory scale, transitioning to large-scale manufacturing processes presents its own set of challenges. Factors such as cost-effectiveness, production efficiency, and regulatory compliance must be carefully considered to ensure the viability of green stabilizers as practical alternatives to traditional stabilizers. Long-term stability testing is essential to assess the effectiveness of green stabilizers under various storage conditions and environmental factors. However, conducting comprehensive stability studies over extended periods can be time-consuming and resource-intensive. Researchers must develop robust testing protocols and analytical methods to accurately evaluate the long-term stability and reliability of NE-based propellants stabilized with green stabilizers. By overcoming these challenges will be essential for the successful implementation of green stabilizers in NE-based propellants and advancing towards more sustainable propulsion technologies.

This challenge can be effectively mitigated through the exploration of various promising green stabilizers, which not only enhance stability but also exhibit superior compatibility with propellant formulations. Understanding how these stabilizers interact with other propellant components could also help in designing more effective stabilizer systems. Additionally, it's crucial to find ways to make the production of green stabilizers more efficient and cost-effective for large-scale industrial use. This might mean refining current production methods or coming up with new techniques to ensure cost-effective and efficient production of green stabilizers on a large scale. Moreover, continued research into long-term stability testing protocols and analytical methods will be essential to assess the performances and reliability of NE-based propellants stabilized with green stabilizers under various storage conditions and environmental factors. This includes exploring advanced analytical techniques and predictive modeling approaches to accurately predict the stability and performance of green-stabilized propellants over extended periods. The toxicological properties of green stabilizers, as well as their decomposition and secondary decomposition products, must be thoroughly examined to ensure that the alternative stabilizer effectively addresses the issues posed by conventional stabilizers containing harmful substances. This assessment is crucial to mitigate adverse impacts on both the environment and human health. Overall, future research should focus on overcoming the identified challenges in this study while also exploring new opportunities for innovation in green stabilizer technology. By advancing our understanding of green stabilizers and their application in NE-based propellants, researchers can contribute to the development of more sustainable and environmentally friendly propulsion technologies.

## 6 Conclusion

In conclusion, the recent advancements in propulsion technology have led to a significant shift towards the development of green propellants. NE are commonly used in solid propellants but exhibit chemical instability, necessitating the use of stabilizers to ensure safety and performance. This study highlights the importance of exploring green stabilizers as alternatives to conventional stabilizers, considering their adverse environmental impacts. The comprehensive review presented various green stabilizers used in propellants and discussed their effects on stability and shelf-life performance. Additionally, the paper examined the stabilization mechanisms of green stabilizers to mitigate decomposition reactions and ensure long-term storage stability. Furthermore, the exploration of natural products, inorganic compounds, and synthetic derivatives as potential green stabilizers holds promise for mitigating environmental hazards while ensuring the reliability and performance of NE-based propellants. Recent advancements in stabilizer technology demonstrate that green stabilizers can offer comparable or even superior performance to conventional stabilizers without the associated health and environmental risks. However, there are still challenges to overcome, including optimizing the performance of green stabilizers, scaling up production for industrial use, and conducting comprehensive stability testing. Future research directions should focus on addressing these challenges while continuing to innovate and explore novel stabilizer materials and formulations. Overall, the insights provided in this study contribute to ongoing efforts in developing safer and more sustainable propellant technologies, addressing both environmental concerns and the need for reliable performance.

## CRediT authorship contribution statement

**Siti Nor Ain Rusly:** Writing – original draft, Visualization, Investigation, Conceptualization. **Siti Hasnawati Jamal:** Writing – review & editing, Validation, Supervision, Resources, Methodology, Funding acquisition, Conceptualization. **Alinda Samsuri:** Writing – review & editing, Visualization, Validation, Project administration. **Siti Aminah Mohd Noor:** Writing – review & editing, Visualization, Validation, Project administration, Conceptualization. **Khoirul Solehah Abdul Rahim:** Writing – review & editing, Visualization, Conceptualization.

## Data availability statement

All data required to support this study is already mentioned in the manuscript.

## Declaration of competing interest

The authors declare that they have no known competing financial interests or personal relationships that could have appeared to influence the work reported in this paper.
